# Visual rigid laryngoscope *versus* Macintosh laryngoscope using low-dose relaxants in thyroid surgery

**DOI:** 10.7717/peerj.21893

**Published:** 2026-10-30

**Authors:** Weihuang Qiu, Tingjie Wang, Lijun Weng, Menglu Shi, Cailing Guo, Binmei Yu, Xianzhong Lin, Bo Lin, Youguang Gao

**Affiliations:** 1 Department of Anesthesiology, Anesthesiology Research Institute, the First Affiliated Hospital, Fujian Medical University, Fuzhou, Fujian, China; 2 The First School of Clinical Medicine, Fujian Medical University, Fuzhou, Fujian, China; 3 Department of Anesthesiology, National Regional Medical Center, Binhai Campus of the First Affiliated Hospital, Fujian Medical University, Fuzhou, Fujian, China

**Keywords:** Tracheal intubation, Visual rigid laryngoscope, Macintosh laryngoscope, Low-dose relaxants, Thyroid surgery, Intraoperative neuromonitoring

## Abstract

**Objective:**

This study evaluated the safety and efficacy of the Macintosh laryngoscope *versus* the visual rigid laryngoscope (VRL) for tracheal intubation under a low-dose muscle relaxant protocol in thyroid surgery, to provide clinical evidence for the selection of optimal intubation tools for this setting.

**Methods:**

In this single-center, randomized controlled trial, two experienced anesthesiologists performed tracheal intubation using either the Macintosh laryngoscope (Mac group, *n* = 47) or VRL (VRL group, *n* = 45) in patients undergoing elective thyroid surgery with Intraoperative neuromonitoring (IONM). The primary outcome was intubation time. Secondary outcomes included first-attempt intubation success rate, hemodynamic changes during the peri-intubation period, laryngoscopic view graded by the Cormack-Lehane classification, and intubation-related complications.

**Results:**

The intubation time was significantly shorter in the VRL group (mean ± SD, 33.27 ± 8.36 s) than in the Mac group (52.19 ± 9.83 s) (*P* < 0.001). The intubation success rate and heart rates did not differ between groups (*P* > 0.05). The mean arterial pressure (MAP) at intubation and the amplitude of MAP increase during intubation was lower in Group VRL than in Group Mac (*P* < 0.005). No statistically significant differences were observed between the two groups in peripheral oxygen saturation (SpO_2_), Cormack-Lehane classification, or intubation-related complications (*P* > 0.05).

**Conclusion:**

For thyroid surgery using a low-dose muscle relaxant protocol to enable IONM, the VRL provides significantly shorter intubation time and better hemodynamic stability at intubation compared with the Macintosh laryngoscope. No significant differences were observed between the two devices in intubation success rate, laryngoscopic view, or intubation-related complications.

## Introduction

Recurrent laryngeal nerve (RLN) injury remains a critical complication of thyroid surgery. Intraoperative neuromonitoring (IONM) is a technique that continuously monitors the electromyographic activity of the recurrent laryngeal nerve during thyroid surgery and is now widely adopted to prevent RLN injury (Wojtczak et al., 2024). However, the use of neuromuscular blocking agents (NMBAs) can diminish the electromyography (EMG) signals derived from IONM, potentially leading to false-negative results (Lu et al., 2017). In addition, reversal of neuromuscular blockade with sugammadex after tracheal intubation may lead to the return of muscle activity, including patient movement or coughing in response to surgical stimulation, which can interfere with the surgical procedure. The current standard protocol for neuromuscular blockade management in IONM involves a single induction dose of a non-depolarizing NMBA (such as 0.5 mg/kg rocuronium or atracurium) to facilitate tracheal intubation, allowing spontaneous recovery of neuromuscular transmission (Chu et al., 2009). This low-dose relaxant strategy, however, inherently increases the difficulty of tracheal intubation: reduced neuromuscular blockade leads to incomplete relaxation of the laryngeal and pharyngeal muscles, which can result in suboptimal intubating conditions, including incomplete vocal cord abduction and increased resistance during laryngoscopy (Li et al., 2021; Lu et al., 2011). Therefore, selecting an appropriate intubation device under low-dose NMBA conditions is critical. To date, no published study has compared the visual rigid laryngoscope (VRL) with the Macintosh laryngoscope for tracheal intubation in this specific setting. We therefore conducted this randomized controlled trial to compare the two devices, with intubation time as the primary outcome.

The Macintosh laryngoscope has been widely regarded as the standard instrument for direct laryngoscopy for over 60 years and remains the most commonly used device for tracheal intubation worldwide (Ranieri Junior & Nascimento Junior, 2021; Suzuki & Iwasaki, 2008). Advances in visualization technology have led to the widespread clinical adoption of the visual rigid laryngoscope, a video-based optical stylet consisting primarily of a lens, camera, display screen, and light source. Compared with the Macintosh laryngoscope, the VRL requires less lifting force and tongue displacement, and its indirect visualization capability reduces the need for a full Cormack-Lehane grade I view of the glottis. These features make it particularly suitable for patients with obesity, severely restricted mouth opening, or limited head and neck mobility (Nam et al., 2019; Vitkovic et al., 2020).

To date, no published study has compared the visual rigid laryngoscope and the Macintosh laryngoscope for tracheal intubation in thyroid surgery using a low-dose muscle relaxant protocol for IONM. We hypothesized that the visual rigid laryngoscope would be superior to the Macintosh laryngoscope in terms of intubation time (primary outcome) and would provide better hemodynamic stability during intubation as a secondary outcome. We therefore conducted this randomized controlled trial to compare the two devices, with the aim of providing clinical evidence to guide the selection of the most effective and safe laryngoscopic technique for this specific clinical scenario.

## Materials & Methods

### Trial design

This was a parallel-group, prospective, randomized controlled trial.

### Participants and eligibility criteria

This study was conducted at the First Affiliated Hospital of Fujian Medical University between May 10, 2022, and May 31, 2023. The study was approved by the Ethics Committee of the First Affiliated Hospital of Fujian Medical University (MRCTA, ECFAH of FMU[2020]381) and registered with the China Clinical Trial Registry (Registration No. ChiCTR2000038945). This manuscript adheres to the applicable CONSORT guidelines.

Inclusion criteria were: (1) American Society of Anesthesiologists (ASA) physical status I or II; (2) age 18 to 65 years; and (3) scheduled for elective thyroid surgery requiring intubation under a low-dose relaxant protocol for intraoperative neuromonitoring. Exclusion criteria included: (1) body mass index (BMI) > 30 kg/m^2^; (2) abnormal cervical spine; (3) throat disease; (4) anticipated difficult airway intubation; and (5) severe hearing or comprehension problems, or inability to cooperate and provide informed consent. Predictors of difficult intubation were Mallampati grade IV, an interincisal distance < three cm, restricted neck movement < 90°, and a thyromental distance < six cm. A history of obstructive sleep apnoea (OSAHS) alone was not considered sufficient to define an anticipated difficult airway; patients with OSAHS were excluded only if they also met one or more of the above anatomical predictors. Seven patients had a documented diagnosis of OSAHS but were not excluded because they had no additional anatomical predictors of difficult intubation. All participants provided written informed consent after receiving a full explanation of the study procedures. A total of 92 patients were enrolled in the study.

### Intraoperative management

All patients fasted for 8 h and abstained from clear liquids for 6 h preoperatively. Upon entering the operating room, patients received standard monitoring, including three-lead electrocardiography, non-invasive blood pressure measurement, and pulse oximetry. Routine peripheral venous access was established for fluid administration, and invasive arterial blood pressure was monitored *via* radial artery cannulation. A multifunctional monitor was used to continuously track invasive arterial pressure, pulse oximetry, non-invasive blood pressure, and inspired oxygen concentration.

Prior to anesthesia induction, all patients received preoxygenation and denitrogenation *via* a face mask. After 3 min of adequate preoxygenation with 100% oxygen, anesthesia was induced with the sequence: lidocaine (40 mg), fentanyl (three µg/kg), propofol (two mg/kg), and rocuronium (0.3 mg/kg). Endotracheal intubation was initiated 120 to 150 s after the completion of rocuronium administration. Tracheal intubation was performed using either a Macintosh laryngoscope or a visual rigid laryngoscope (Fig. 1), as assigned by randomization, with a seven mm EMG endotracheal tube. The tube was positioned such that its lateral electrodes made contact with the vocal cords, and was then secured in place.

**Figure 1:**
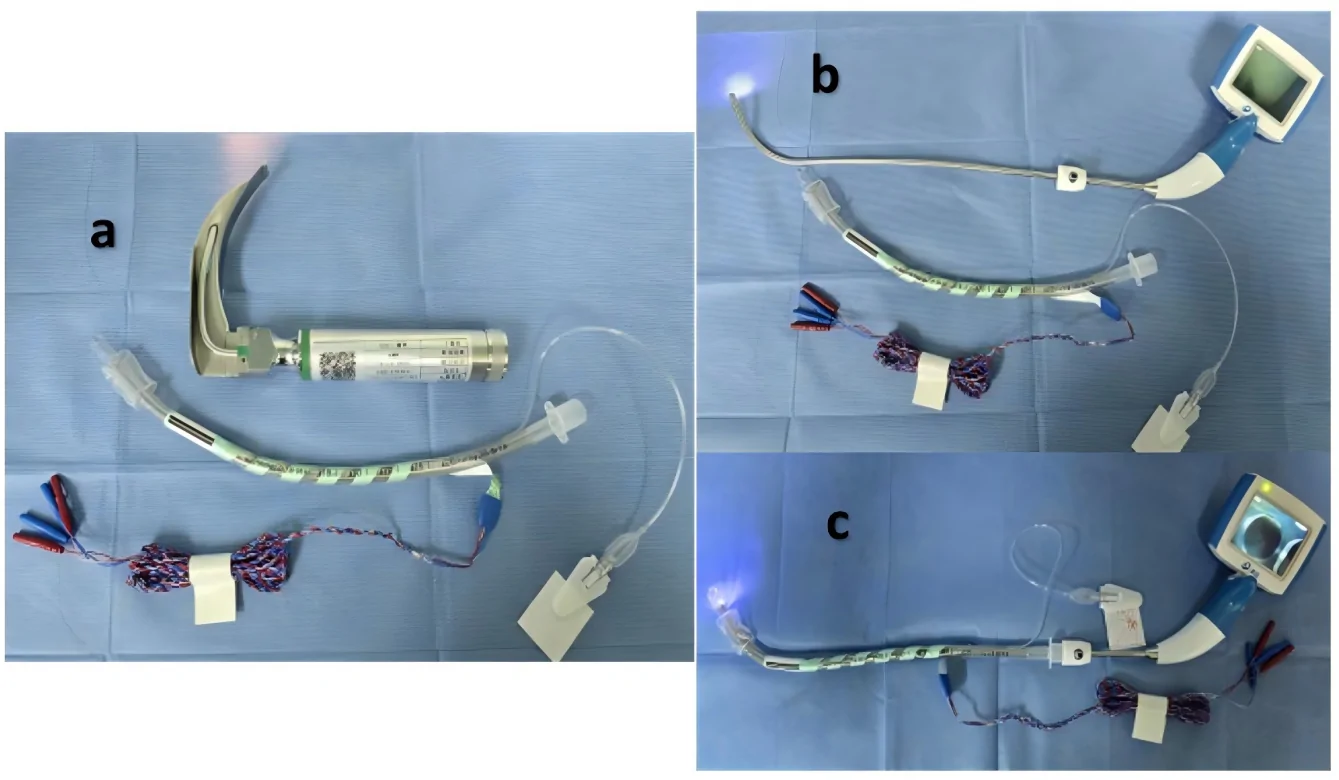
The picture of the laryngoscope and EMG endotracheal tube. (A) Macintosh laryngoscope and EMG endotracheal tube; (B, C) visual rigid laryngoscope and/with EMG endotracheal tube.

All intubations were performed by two anesthesiologists, each with more than three years of clinical anesthesia experience and over 200 clinical uses of each laryngoscope type. In the Mac group, tracheal intubation was performed using a Macintosh laryngoscope (5456D; SHUCMAN, Netanya, Israel). In the VRL group, a visual rigid laryngoscope (TRS-K2; Zhejiang UE Medical Corp, Zhejiang, China) was inserted along the pharyngeal midline with a preloaded endotracheal tube, a jaw lift was performed if needed to facilitate insertion. No other intubation devices were permitted during the study procedure, although external laryngeal manipulation was allowed at any time with both devices.

Intubation failure was defined as failure to complete intubation within 180 s or after three attempts. The same anesthesiologist was permitted a maximum of three intubation attempts per patient. If intubation attempts failed, an alternative intubation method was used, and the event was recorded.

Following successful intubation, the patient was connected to an anesthesia machine for volume-controlled ventilation with an air-oxygen mixture at a 1:1 flow ratio. Tidal volume was set at 6–8 ml/kg, with an inspiratory-to-expiratory ratio of 1:1.5 to 1:2, respiratory rate of 12–20 breaths per minute, and target end-tidal carbon dioxide partial pressure (PetCO_2_) of 35–45 cmH_2_O. Peak inspiratory pressure (Ppeak) was maintained below 30 cmH_2_O. Anesthesia was maintained with inhaled sevoflurane at a concentration of 1–2%, supplemented by intravenous infusions of propofol at 40–60 µg/kg/min and remifentanil at 0.1–0.2 µg/kg/min to ensure stable vital signs. The infusion rates of these maintenance agents were adjusted in response to fluctuations in the patient’s physiological parameters.

The primary outcome was intubation time, defined as the interval from insertion of the laryngoscope into the oral cavity to confirmation of correct endotracheal tube placement *via* positive capnography. Intubation time was selected as the primary outcome because its magnitude under the specific conditions of low-dose relaxant use with an EMG tube had not been previously quantified. The theoretical advantages of the VRL—while plausible—needed direct measurement in this specific clinical context to inform practical decision-making. Secondary outcomes included the first-attempt success rate of intubation, hemodynamic changes during the peri-intubation period, intubation conditions assessed *via* the Cormack-Lehane classification, and intubation-related complications. Heart rate and arterial pressure were recorded at five time points: before anesthesia induction (T1), before intubation (T2), at the time of intubation (T3), and 3 min (T4) and 5 min (T5) after intubation. Intubation-related complications were documented, including mucosal damage (identified by the presence of blood), lip or dental trauma, coughing/bucking during intubation, and postoperative nausea, vomiting, or sore throat occurring within 24 h after surgery. Follow-up personnel were blinded to group assignments.

### Sample size calculation

The minimum sample size was calculated based on the primary outcome of intubation time. In a preliminary trial involving 20 patients, the mean intubation time with the Macintosh laryngoscope was 48 ± 11 s, and use of the visual rigid laryngoscope reduced intubation time by 15%. Setting *α* at 0.05 and power (1 − *β*) at 0.8, the calculation indicated that 45 participants per group were required. Anticipating a 10% loss to follow-up rate, we determined a final sample size of 50 patients per group. Although the final enrollment (92 patients) fell slightly short of the planned target of 100, both groups (47 in the Mac group and 45 in the VRL group) met or exceeded the minimum requirement of 45 patients per group, and the effective sample size therefore remained sufficient for the primary analysis.

### Randomization (random number generation, allocatiaon concealment, implementation)

Computer-generated randomization numbers in sealed opaque envelopes assigned patients in a 1:1 ratio to either the Macintosh laryngoscope (Group Mac) or the visual rigid laryngoscope (Group VRL). Following successful induction of anaesthesia and confirmation of mask ventilation, the envelope was opened immediately in the presence of other staff members.

### Blinding

All intubations were performed by one of two experienced anesthesiologists (each with >3 years of experience and >200 uses of each device); neither was informed of the device assignment until the patient arrived in the operating room. A separate researcher, blinded to the study’s purpose, recorded all data.

### Statistical methods

All data were analyzed using a two-sample *t*-test power analysis (Zar & Hall, 1999), and all statistical analyses were performed using R version 4.3.1 (R Core Team, 2023). Quantitative results are reported as the median with interquartile range for non-normally distributed data or as the mean ± standard deviation (SD) for normally distributed data. Categorical data are summarized as percentages. Group differences for quantitative variables were assessed with the Wilcoxon–Mann–Whitney test for non-normal distributions or Student’s *t*-test for normal distributions. Comparisons across multiple time points were conducted using repeated-measures ANOVA for normally distributed data. Count data are expressed as ratios or percentages and were analyzed with the chi-square test or Fisher’s exact test. A *P*-value < 0.05 was considered statistically significant.

## Results

A total of 100 patients met the initial inclusion criteria. Of these, six were excluded (four due to a history of difficult intubation and two due to refusal to participate), and two withdrew before surgery. Consequently, 92 patients were ultimately included in the final analysis (47 in the Mac group, 45 in the VRL group) (Fig. 2).

**Figure 2:**
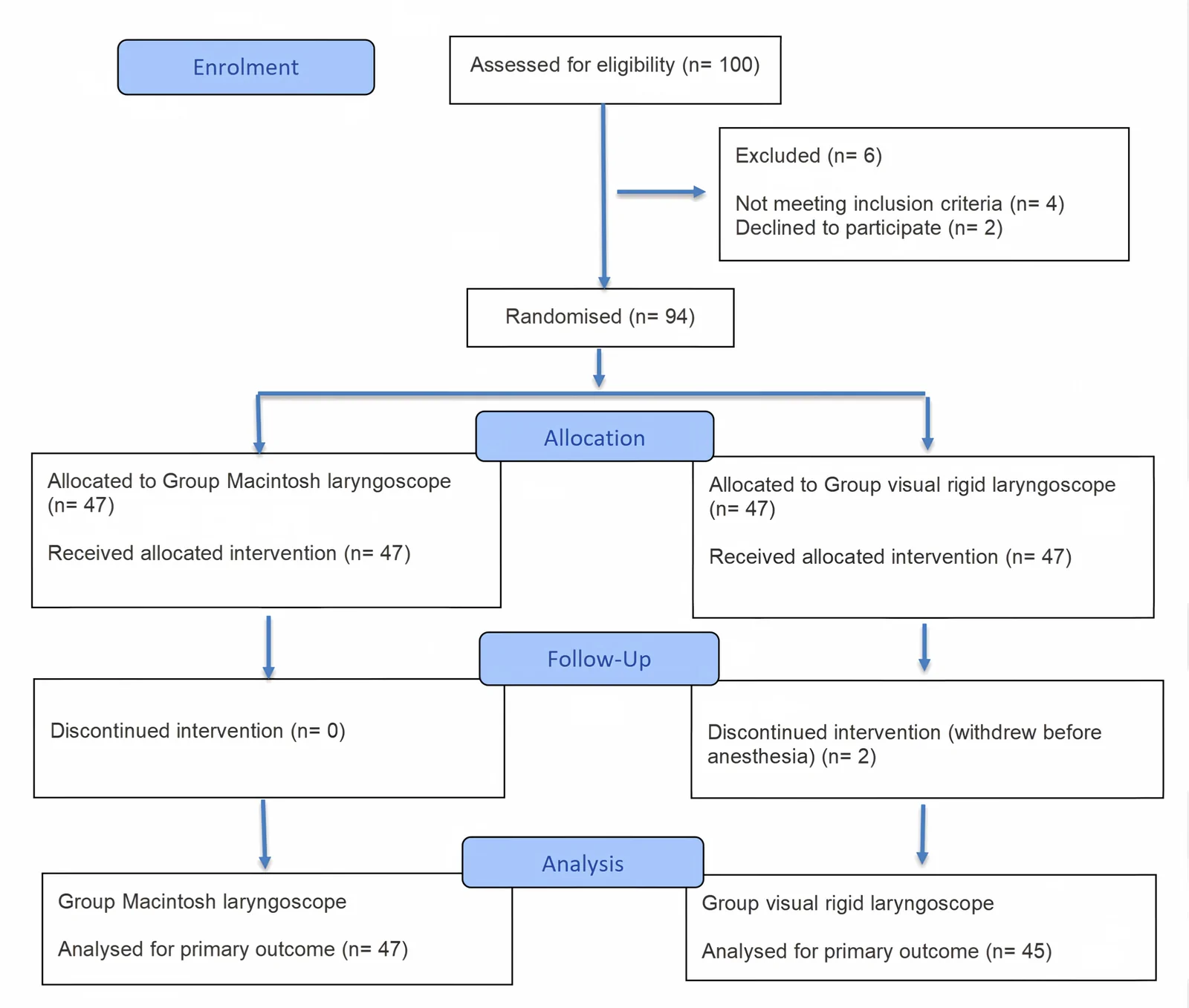
CONSORT 2025 flow diagram. Flow diagram of the progress through the phases of a randomised trial of two groups (that is, enrolment, intervention allocation, follow-up, and data analysis).

Airway-related variables and demographic characteristics of the two groups appear in Table 1. In the Mac group, one hypertensive patient was taking valsartan and metoprolol, one was taking valsartan, one was taking amlodipine, and four were not receiving medication for hypertension control (despite having a documented history of hypertension). In the VRL group, one hypertensive patient was taking irbesartan and metoprolol, one was taking ramipril and amlodipine, two were taking amlodipine, and two were not receiving antihypertensive medication (despite having a documented history of hypertension).

**Table 1:** Comparisons of demographic characteristics and airway-related variables between the 2 groups.

	Group Mac (*n* = 47)	Group VRL (*n* = 45)
Age (y)	45.87 (20.00–66.00)	43.18 (20.00–71.00)
Sex		
Male, *n* (%)	11 (23.40)	14 (31.11)
Female, *n* (%)	36 (76.60)	31 (68.89)
Height (cm)	161.09 (145.00–173.00)	163.44 (150.00–180.00)
Weight (kg)	60.61 ± 10.02	63.3 ± 11.59
Body mass index (kg/m^2^)	23.24 ± 2.92	23.52 ± 2.71
Thyromental distance (cm)	7.27 (6.00–8.00)	7.44 (6.00–10.00)
Interincisor distance (cm)	5.46 (3.50–7.50)	5.82 (4.00–7.50)
Neck circumference (cm)	33.72 ± 5.06	34.13 ± 5.25
Limited range of motion of temporomandibular joint and head and neck joint, *n* (%)	0, (0)	0, (0)
Tracheal compression, *n* (%)	0, (0)	0, (0)
Missing incisors, *n* (%)	1 (2.13)	3 (6.67)
Mallampati grade (1/2/3/4)	32/14/1/0	28/17/0/0
ASA grade (1/2)	10/37	18/27
Comorbidities, *n* (%)		
Hypertension	7, (14.89)	6, (13.33)
Sinus bradycardia	2, (4.25)	1, (2.22)
Atrial fibrillation	1, (2.13)	0, (0)
Frequent ventricular premature	1, (2.13)	0, (0)
Interstitial pneumonia	1, (2.13)	0, (0)
OSAHS	3, (6.38)	4, (8.89)

**Notes.**

Values are the mean ± standard deviation, the number (proportion) or median (min–max).

Group Mac Tracheal intubations were performed using Macintosh laryngoscope group

Group VRL Tracheal intubations were performed using Visual rigid laryngoscopy group

ASA American Society of Anesthesiologists OSAHS Obstructive Sleep Apnea-Hypopnea Syndrome

Intubation time was significantly shorter in the VRL group (mean ± SD, 33.27 ± 8.36 s) than in the Mac group (52.19 ± 9.83 s), with a between-group difference of −18.92 s (95% CI [−22.71 to −15.13 s]) (Fig. 3).

**Figure 3:**
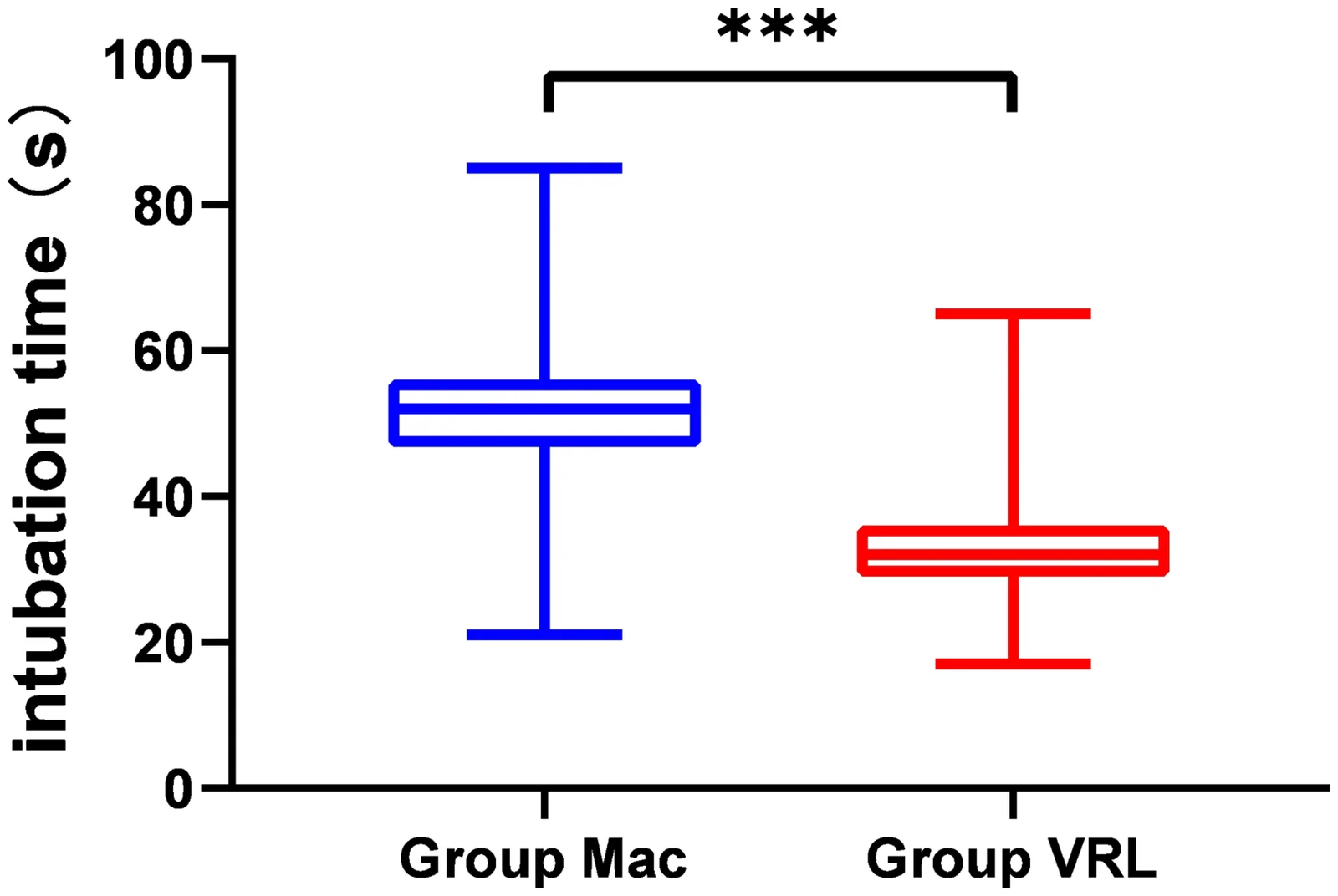
Comparison of intubation time between the two groups. The box spans the interquartile range (25th–75th percentiles), the horizontal line inside each box indicates the median value. Whiskers represent the observed minimum and maximum values. Group Mac, Macintosh laryngoscope group. Group VRL, Visual rigid laryngoscopy group. ***P < 0.001.

In the Group Mac, 46 patients were intubated on the first attempt, while 1 required a second attempt. All 45 patients in the Group VRL were intubated successfully on the first attempt. The first-attempt success rate was 97.87% for the Group Mac and 100% for the Group VRL, with no statistical significance (risk difference 2.13%; 95% CI [−2.90 to 7.16]; *P* = 1.000).

There were no statistically significant between-group differences in heart rates at any time point (all *P* > 0.05; Fig. 4A). Heart rate increased significantly during intubation in both groups (T3 compared with T2; Mac group: difference between means ± SEM 9.723 ± 2.594, 95% CI [4.571–14.88], *P* < 0.001; VRL group: difference between means ± SEM 5.978 ± 2.716, 95% CI [0.504–11.45], *P* = 0.033; Fig. 4A). The magnitude of heart rate increase during intubation (T3 minus T2) did not differ significantly between the two groups (*P* = 0.100; Fig. 4C).

**Figure 4:**
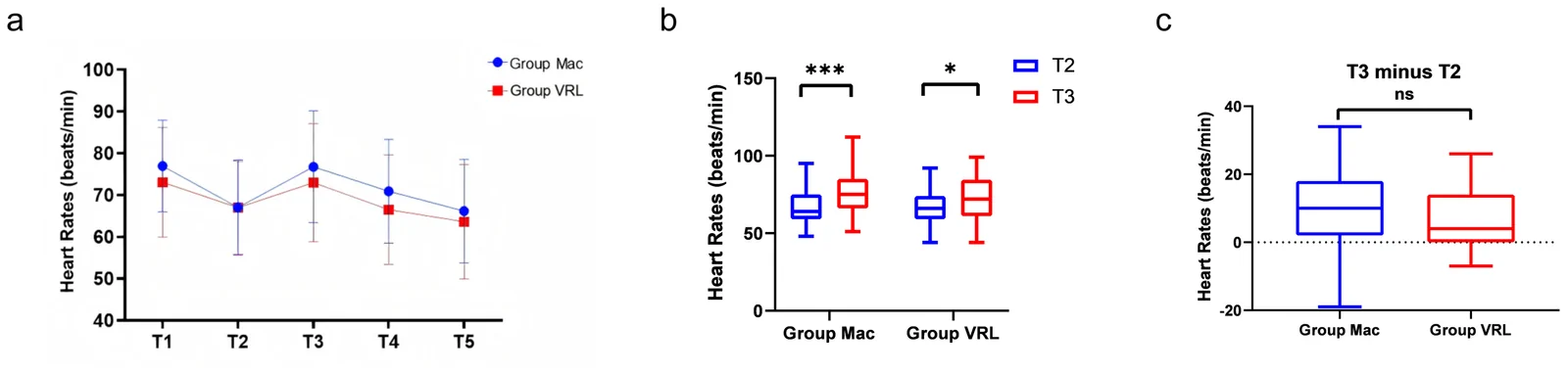
Comparison of heart rate between the Group Mac and Group VRL. (A) Mean Heart Rates (HR) changes across different time-points during intubation, P (T1) = 0.171; P (T2) = 0.994; P (T3) = 0.183; P (T4) = 0.097; P (T5) = 0.333. (B) HR increased during intubation (T3 than T2) in both groups, ***P (Group Mac) < 0.001, *P (Group VRL) = 0.033. (C) The increase of HR in intubation (Compare the values of T3 minus T2) between two groups, *P* = 0.100. Group Mac, Macintosh laryngoscope group. Group VRL, Visual rigid laryngoscopy group. T1, Before anesthesia induction; T2, Before intubation; T3, at intubation; T4, 3 min after intubation; T5, 5 min after intubation. ns, not significant.

Changes in MAP (mean) at T1, T2, T4, T5 phases of intubation was not significant difference (all *P* > 0.05) (Fig. 5A), MAP of the visual rigid laryngoscope group was lower than that of the Macintosh laryngoscope group at intubation (comparison of two groups at T3, *P* = 0.003) (Fig. 5B). MAP increased significantly from T2 to T3 in both groups (both *P* < 0.001; Fig. 5C). The magnitude of MAP increase during intubation (T3 minus T2) was significantly more pronounced in the Mac group than in the VRL group (mean difference −10.53 mmHg, 95% CI [−16.65 to −4.414 mmHg]; *P* < 0.001; Fig. 5D).

**Figure 5:**
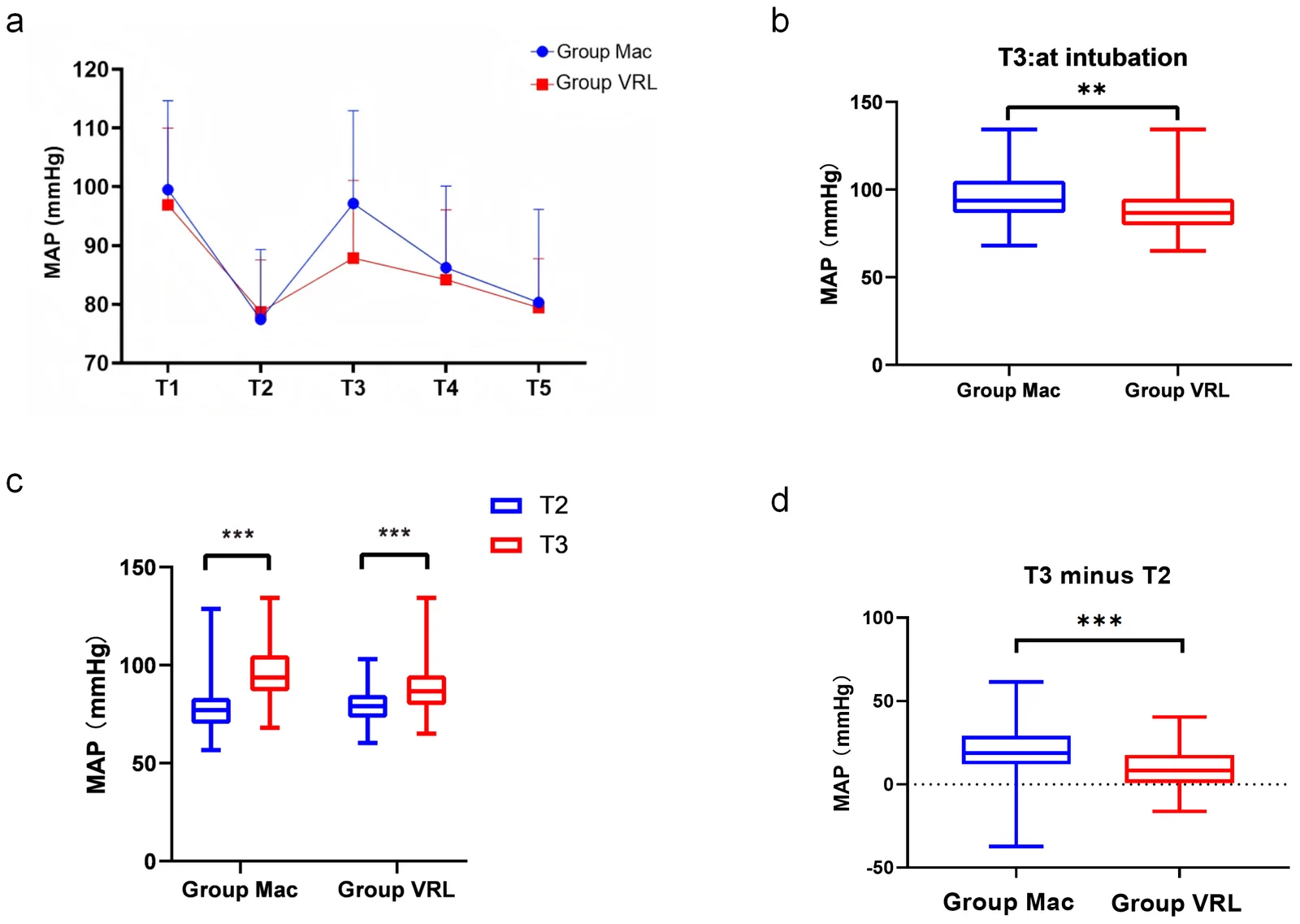
Comparison of mean arterial pressure (MAP) (mean) between the Group Mac and Group VRL. (A) Changes in MAP (mean) at different phases of intubation; P (T1) = 0.39; P (T2) = 0.577; P (T3) = 0.003; P (T4) = 0.451; P (T5) = 0.195. (B) MAP in the Group Mac and Group VRL at T3; **P = 0.003 (median, IQR). (C) MAP during intubation (T3 *vs* T2) in both groups; both ***P < 0.001 (median, IQR). (D) The increase of MAP during intubation (T3 minus T2) between the two groups; ***P < 0.001 (median, IQR). Group Mac, Macintosh laryngoscope group. Group VRL, Visual rigid laryngoscopy group. T1, Before anesthesia induction; T2, Before intubation; T3, at intubation; T4, 3 min after intubation; T5, 5 min after intubation.

No statistically significant between-group differences were observed in peripheral oxygen saturation (SpO_2_) at any time point (all *P* > 0.05; Fig. S1). The Cormack-Lehane classification also did not differ significantly between groups (*P* = 0.753). During intubation, four patients in the Mac group developed coughing/bucking, but the overall incidence of intubation-related complications showed no statistically significant between-group difference (*P* = 0.117). No postoperative adverse events were reported in either group (Table 2).

**Table 2:** Comparison of Cormack-Lehane classification and intubation-related complications between the two groups.

	Group Mac (*n* = 47)	Group VRL (*n* = 45)	*P* value
Cormack-Lehane classification, (1/2/3/4)	32/15/0/0	32/13/0/0	0.753
Intubation-related complications, *n* (%)	4 (8.51)	0 (0)	0.117
Postoperative complaints, *n* (%)	0 (0)	0 (0)	NA

**Notes.**

Values are the number (proportion).

Group Mac Macintosh laryngoscope group

Group VRL Visual rigid laryngoscopy group

## Discussion

In this study, we found that tracheal intubation with a visual rigid laryngoscope under a low-dose muscle relaxant protocol in thyroid surgery with IONM was associated with a significantly shorter intubation time compared with the Macintosh laryngoscope. Additionally, MAP at the time of intubation and the amplitude of MAP increase during intubation was significantly lower in the VRL group than in the Mac group. No significant between-group differences were observed in intubation success rate, intubation conditions, or intubation-related complications.

The shorter intubation time observed with the VRL relative to the Macintosh laryngoscope may be attributed to several procedural factors. First, pre-intubation preparation for the VRL involves mounting the tracheal tube onto its rigid stylet, which eliminates the time required to advance the tube separately during the intubation attempt. Accordingly, the tube can be advanced immediately once the vocal cords are visualized. In contrast, during tracheal intubation with a Macintosh laryngoscope, the vocal cords must first be exposed with the laryngoscope before the tracheal tube can be introduced into the oral cavity and advanced into the trachea, a process that inherently takes longer. Second, intubating conditions in patients receiving low-dose muscle relaxants for thyroid surgery are inferior to those achieved with high-dose relaxants (Lu et al., 2011). The large blade size and greater intraoral occupancy of the Macintosh laryngoscope—coupled with the larger-diameter EMG tube and suboptimal soft tissue relaxation under low-dose muscle relaxants—may restrict maneuverability and increase resistance during tube passage, particularly when laryngeal exposure is suboptimal. Third, the EMG endotracheal tube used for IONM has a larger outer diameter and inflated cuff than conventional endotracheal tubes, which increases traumatic stimuli during intubation and extubation and requires more intraoral space during placement. This presents a distinct disadvantage for the Macintosh laryngoscope.

Reducing intubation time is clinically significant for patients. First, shorter intubation duration decreases the time of laryngoscope manipulation in the oral and pharyngeal cavities, thereby lowering the risk of associated trauma. Second, prolonged laryngoscope use amplifies the procedural stress response (Hirabayashi et al., 1998). The increase in arterial pressure associated with laryngoscopy typically begins within 5 s of instrument insertion, peaks within 1–2 min, and returns to baseline levels within 5 min (Sulaiman et al., 2012). While a modest reduction in intubation time may not affect complication rates in elective surgery patients with adequate preoperative preparation and no significant comorbidities, our results have important reference value for intubation under low NMBA dosing or insufficient anesthesia depth.

In this study, MAP during intubation was statistically significantly lower with the VRL than with the Macintosh laryngoscope, and the magnitude of MAP increase during intubation was also smaller in the VRL group. Although this difference in MAP reached statistical significance, its clinical significance is uncertain and likely modest, as both groups maintained MAP within clinically acceptable ranges. This outcome is predictable, as the shorter intubation time with the VRL results in a reduced pressor response and less stimulation of the sympathetic nervous and cardiovascular systems. This finding has important clinical relevance for laryngoscope selection in patients requiring greater hemodynamic stability during intubation. Tracheal intubation induces a stress response primarily for two reasons (Hirabayashi et al., 1998), First, neural reflexes are triggered by stimulation of pharyngeal and laryngeal tissues during laryngoscope insertion and elevation. Second, the trachea itself is stimulated following tube placement, with direct tracheal stimulation appearing to be the primary cause of the associated hemodynamic changes (Kanaide et al., 2003). Furthermore, unlike the Macintosh laryngoscope, the VRL does not require significant elevation of the epiglottis to expose the glottis. Its rigid core structure and integrated video lens facilitate guidance of the catheter under the epiglottis and through the vocal cords, allowing the tube to be inserted into the trachea with minimal manipulation once the epiglottis and glottis are visualized. This reduced manipulation further decreases procedural stimulation. In a previous similar study conducted by our team in elderly patients, we found no difference in hemodynamic effects between a video laryngoscope and the VRL (Weng et al., 2024). This discrepancy in findings is likely due to the different comparator devices used in the two studies, which limits direct comparability. We speculate that the difference between the Macintosh laryngoscope and the VRL may be greater than that between a video laryngoscope and the VRL, resulting in greater stimulation intensity and a more pronounced hemodynamic response.

In this study, there was no significant between-group difference in the first-attempt success rate of tracheal intubation. One patient in the Mac group experienced inadvertent esophageal intubation on the first attempt, which was immediately recognized and corrected with a successful second intubation. For well-trained operators, Abdullah et al. (2013) found no difference in the success rate of tracheal intubation using either the Bonfils intubation fiberscope or the McCoy laryngoscope. Consistent with this finding, we observed that under stable conditions, the intubation success rate does not differ significantly between different intubation devices when used by experienced operators. The anesthesiologists in this study were well-trained and highly experienced in intubation, and successful intubation was not technically challenging in this cohort of patients with non-difficult airways. Furthermore, we excluded patients with predicted difficult intubation from the outset. Further research is needed to evaluate the performance of these two laryngoscopes in patients with difficult airways. Similar findings were reported by Ji et al. (2018), who compared low-dose (0.3 mg/kg) with conventional-dose (0.6 mg/kg) rocuronium for intubation using a video laryngoscope. Although low-dose rocuronium resulted in significantly worse overall intubation conditions, the first-attempt success rate and intubation time did not differ between the two groups. The authors concluded that when using a video laryngoscope, low-dose rocuronium may provide clinically acceptable laryngeal muscle relaxation. Taken together, these results suggest that in the setting of low-dose neuromuscular blockade, the use of an appropriate video-assisted device can compensate for suboptimal muscle relaxation to achieve efficient intubation. Further research is needed to evaluate the performance of these two laryngoscopes in patients with difficult airways.

In this study, there were no significant between-group differences in SpO_2_, Cormack-Lehane classification, or intubation-related complications. A larger sample size may be required to detect statistically significant differences in intubation conditions and related complications between the two devices.

This study has several limitations. First, the performance of the specific intubation devices used in this study may differ from other models of Macintosh or video laryngoscopes. Second, due to the inherent differences between the two intubation techniques, only the patients and postoperative follow-up personnel could be blinded; the intubating anesthesiologist, intubation observer, and data collector could not be blinded to group assignment. Third, because patients with predictors of difficult intubation were excluded, our results cannot be extrapolated to difficult airway management scenarios. Fourth, while patients were randomized and the prevalence of hypertension did not differ significantly between groups, the hemodynamic response to tracheal intubation may be influenced by the specific antihypertensive medications used by individual patients. Fifth, while this study investigated intubation under a low-dose relaxant protocol for thyroid surgery with IONM, we did not include a control group receiving a full dose of muscle relaxants to determine whether NMBA dose was the primary driver of observed differences in intubation time and hemodynamic changes. Sixth, the two anesthesiologists who performed all intubations were highly experienced with both devices (each with >200 uses per device). While this standardization minimized operator-related variability and strengthened internal validity, it may limit the generalizability of our findings to settings where clinicians are less experienced with the VRL or Macintosh laryngoscope.

## Conclusions

In thyroid surgery using a low-dose muscle relaxant protocol to enable IONM, the visual rigid laryngoscope provides a significantly shorter intubation time and better hemodynamic stability at the time of intubation compared with the Macintosh laryngoscope. No significant differences were observed between the two devices in terms of intubation success rate, laryngoscopic view, or intubation-related complications.

##  Supplemental Information

10.7717/peerj.21893/supp-1 Supplemental Information 1 Raw data

10.7717/peerj.21893/supp-2 Supplemental Information 2 Supplemental Figures

10.7717/peerj.21893/supp-3 Supplemental Information 3 CONSORT checklist

## Additional Information and Declarations

### Data Availability

The following information was supplied regarding data availability: The data is available in the Supplemental Files.

### Clinical Trial Registration

The following information was supplied regarding Clinical Trial registration: No. ChiCTR2000038945.
