# Supplementary material for: Visual rigid laryngoscope versus Macintosh laryngoscope using low-dose relaxants in thyroid surgery
Source: PeerJ. 2026 Oct 30;14:e21893. doi: 10.7717/peerj.21893 (PMC13726576; doi:10.7717/peerj.21893)
Supplement: Supplementary file 1 [file peerj-14-21893-s002.docx]

**Supplementary Fig 1.Changes in SpO2 (mean) at different phases of intubation.** There was no significant difference in the mean SpO2 between the Macintosh laryngoscope group and the visual rigid laryngoscope group during different phases. P (T1) = 0.9465; P (T2) = 0.9243; P (T3) = 0.9266; P (T4) = 0.9426; P (T5) = 0.8703. T1: Before anaesthesia induction; T2: Before intubation; T3: at intubation; T4: 3 min after intubation; T5: 5 min after intubation.
